# Low-Dose Baclofen-Induced Encephalopathy in a Healthy Young Adult: Is Baclofen Toxicity Dose-Dependent?

**DOI:** 10.7759/cureus.20499

**Published:** 2021-12-18

**Authors:** Emeka Ibeson, Ifeanyi Nwosu, Thai Donenfeld, Britney Clemen, Ufeh Annabel Ogar, Omosefe E Ogbeifun, Michael Marcelin

**Affiliations:** 1 Internal Medicine, Maimonides Medical Center, Brooklyn, USA; 2 Internal Medicine, Peterborough City Hospital, Peterborough, GBR; 3 Public Health, University of West Florida, Pensacola, USA

**Keywords:** nsaids, renal impairment, drug interaction, encephalopathy, baclofen toxicity

## Abstract

Baclofen is a commonly used medication for managing muscle spasticity with the potential of causing life-threatening adverse effects, including encephalopathy as well as withdrawal symptoms including confusion and hallucinations following abrupt discontinuation. Severe toxicity with baclofen is common in patients with kidney disease, hence the importance of dose reduction and monitoring in patients with renal impairment. This article reports a case of encephalopathy with low-dose baclofen in an otherwise healthy young adult concurrently taking ibuprofen and baclofen and aims to underscore the importance of potential drug interaction and patient education when initiating baclofen.

## Introduction

Baclofen is an FDA approved, centrally acting gamma aminobutyrate acid (GABA) agonist that exerts its action by inhibiting the presynaptic motor neurons and is used for the treatment of spasticity, especially for the symptomatic relief of flexor spasm, clonus, and pain in patients with multiple sclerosis, cerebral palsy, and spinal cord lesions [1].

Severe toxicity is uncommon and more so in patients with renal disease or impairment as the kidneys are primarily responsible for the elimination, with about 60-80% of the medication excreted unchanged [2,3]. Baclofen toxicity may result in respiratory depression, hypotonia, hyporeflexia, cardiac arrhythmias, bradycardia, seizures, and coma. Studies suggested that toxicity is likely dose-dependent and more common at doses greater than 200mg [4,5]. It is worth noting that severe baclofen withdrawal may occur within hours to days of sudden cessation or dose reduction and is mostly associated with altered mental status, hallucinations, worsening spasticity, and fever [6]

The usual maximum daily dose of oral baclofen is 80mg [1], although some patients require up to 120mg/day [7]. Toxicity with high doses is seen in accidental or intentional overdose and patients requiring intrathecal baclofen [3]. However, patients with renal impairment are at greater risk of severe adverse effects even at small doses [8]. Reports of low-dose baclofen-induced encephalopathy in patients without renal disease are scarce. To the best of our knowledge, only a small number of such cases exist in the literature and all are in elderly patients [9-12] with one exception [13]. Other than renal impairment, the risk of severe toxicity and encephalopathy may be increased due to drug interactions, even at low doses. We report a case of low-dose baclofen-induced encephalopathy in an otherwise healthy young patient. Notably, the patient was concurrently taking ibuprofen and baclofen to relieve chronic back pain and had no other identifiable cause of encephalopathy. Thus, we aim to highlight the importance of drug interaction awareness and patient education while initiating baclofen, even for short-term use.

## Case presentation

A 21-year-old Egyptian female with no significant past medical history was brought to the emergency department (ED) by the paramedics with altered consciousness preceded by acute lethargy about eight hours before presentation. Her family reports that she appeared normal earlier that morning before complaining of dizziness in the afternoon, for which she decided to take a nap. She was heard screaming, “I am going to die; I am going to die,” shortly after she had gone for a nap. She became more lethargic, had an episode of vomiting, and the family contacted the emergency services. Assessment by the paramedics noted that she was severely obtunded, confused, and was minimally responsive. Of note, the patient had a transient episode of hallucination two days prior, when she reported feeling snakes crawling over her body. She had reported intermittent headaches for the past two months, for which she self-medicated with ibuprofen (Advil) as required. The patient's family members were exposed to a confirmed case of a coronavirus disease 2019 (COVID-19) patient about a month prior, but no family member ever had symptoms. She had not reported fever, cough, or shortness of breath to the family. She is not known to take any regular medication or hormone therapy and did not endorse cigarettes, alcohol, or recreational drugs. She immigrated from Egypt three years ago and had recently returned from a three-month vacation to Egypt two months before the presentation. She stayed with her family, who lives in a rural town; with notable livestock, including pigs, chickens, and dogs. A history of recent baclofen usage, which she revealed after full recovery within 72 hours of hospitalization, was unknown to the medical team at the presentation. She had commenced baclofen at 5mg three times daily two days before admission to manage back pain. In addition, she reportedly took an extra dose of baclofen, a total of 10mg with ibuprofen, the morning of the presentation as she had missed the previous night-time dose. Initial assessment in the ED showed a patient with normal vital signs, unconscious with a Glasgow Coma Scale score of 8, no neck stiffness or focal neurological deficits. The rest of the systemic examinations were normal. The patient was intubated for airway protection, sedated, and ventilated. The neurology team evaluated the patient, and she was worked up upon arrival to the ED. Differential diagnoses included subarachnoid hemorrhage, infectious meningoencephalitis, acute stroke, seizure disorder, other causes of infectious encephalopathy, including Lyme disease, neurosyphilis, and autoimmune encephalitis. 

Head imaging studies were obtained in the ED. CT angiogram of the head showed no hemorrhage, large vessel occlusion, or significant stenosis, and there was no territorial perfusion mismatch. The MRI brain with IV contrast showed no acute infarction, mass, or abnormal signaling. Initial pertinent laboratory investigations are shown in Table 1 below.

**Table 1:** Initial pertinent laboratory investigations. Estimated glomerular filtration rate (eGFR), cerebrospinal fluid (CSF)

Hematology/Biochemistry	Result	Reference Range
Hemoglobin (g/dl)	12.6	12-16
White blood cells (K/ul)	16.3	4.8-10.8
Platelets (K/ul)	288	150-450
Sodium (mmol/l)	143	135-149
Potassium (mmol/l)	3.9	3.4-4.8
Urea (mg/dl)	12.2	7-21
Creatinine (mg/dl)	0.6	0.3-1.1
eGFR (ml/min/1.73m^2^)	>60	>60
Anion gap (mmol/l)	9	3-11
CSF red blood cell (U/l)	611	0
CSF white blood (U/l)	79	0-10
CSF glucose (mg/dl)	107	40-170
CSF protein (mg/dl)	23	20-100

The liver panel, ammonia, serum alcohol, salicylate, and acetaminophen levels were within normal levels. Coronavirus disease-polymerase chain reaction (COVID-PCR) test was negative. Urinalysis and urine toxicology screen were unremarkable for urinary tract infection and toxins, respectively. Initial lumbar puncture (LP) in ED showed clear cerebrospinal fluid (CSF). Other CSF parameters are shown in Table 1. CSF polymerase chain reaction was negative for common bacterial and viral pathogens, including *Escherichia coli*, *Hemophilus influenza*, *Listeria monocytogenes*, *Neisseria meningitides*, *Streptococcus agalactia*, *Streptococcus pneumonia*, Cytomegalovirus, herpes simplex virus 1-2, human herpesvirus 6, varicella-zoster virus, Parechovirus, and Cryptococcus. Treponemal AB test and HIV screening test were also negative. Given the recent travel to Egypt, we sent West Nile virus and rabies titers which were negative. The patient was admitted to the medical intensive care unit (MICU) for further workup and empirically treated with broad-spectrum antibiotics and antiviral medications. A repeat LP was obtained in the MICU within 24 hours of admission while on empiric treatment due to the initial abnormal cell counts consistent with a probable traumatic tap. The repeat specimen showed normal findings. She was weaned from mechanical ventilation and extubated within 72 hours of admission with complete recovery of consciousness and no neurological deficits. Antimicrobial agents were also discontinued early with the return of negative blood, CSF, and urine cultures as well as negative viral and bacterial PCR studies, and the patient was discharged home. 

## Discussion

Our patient was a healthy young female who recently commenced low-dose baclofen taken concurrently with ibuprofen for symptomatic relief of back pain and subsequently developed severe encephalopathy requiring intubation and mechanical ventilation. Extensive investigation for infectious, metabolic, and structural causes of encephalopathy was negative. The profound adverse effect of baclofen in our patient may be linked to an interaction between baclofen and ibuprofen, which the patient had taken with a slightly higher dose of baclofen. Little is known about the drug interaction between baclofen and ibuprofen other than the increased risk of impaired excretion of baclofen following nonsteroidal anti-inflammatory drugs (NSAIDs)-induced renal injury [14]. Baclofen is mainly excreted by the kidneys unchanged, with only about 15% of absorbed baclofen metabolized in the liver through deamination [14]. Our patient had a normal liver function and no pre-existing liver disease to account for possible impaired metabolism. A similar case of baclofen-induced encephalopathy linked to concurrent use with ibuprofen was reported in the literature. In the case mentioned above, an associated acute kidney injury may have increased the risk of toxicity [15]. The evidence supporting a direct adverse drug interaction between NSAIDs and baclofen in the absence of impaired renal function is not clearly substantiated. Our patient had no history of kidney disease and maintained normal renal function throughout her hospital course.

Baclofen interaction with other medications may increase the risk of severe toxicity even at small doses. Several drugs decrease the excretion of baclofen, increasing its serum level. Some directly increase the risk of baclofen-induced adverse effects, including commonly used medications like loop diuretics, angiotensin-converting enzyme (ACEI), angiotensin-receptor blocker (ARB), opioids, benzodiazepines (BZDs), folic acid, and cyanocobalamin [14]. Encephalopathy with baclofen may be exacerbated by co-administration with BZDs [16], and this may explain the onset of acute encephalopathy in one of the reported cases of toxicity with low-dose baclofen [12]. It is not surprising that most patients who take baclofen for relief of muscle spasms may also be on analgesics, of which NSAIDs and opioids are common. Our patient made a full recovery and volunteered additional drug history upon recovery. Unfortunately, she was not aware of the side effects of baclofen and perhaps may have discontinued medication following the first episode of hallucinations.

## Conclusions

Several factors contribute to the increased risk of baclofen toxicity and thus are not simply a dose-dependent risk. Therefore, clinicians prescribing baclofen or monitoring patients who require long-term baclofen must be aware of various factors that may potentiate toxicity. Renal impairment and drug interactions can increase the risk of severe toxicity even with normal renal function. Since many of the adverse effects of baclofen are life-threatening, patients must be thoroughly counseled about the potential side effects of baclofen where possible and be advised to discontinue medications at the slightest symptom. In addition, as patients can obtain NSAIDs over the counter, they must be educated by clinicians on the potentials of increased risk of toxicity in the setting of NSAID-induced renal impairment. The takeaway lessons from this clinical case include severe baclofen toxicity may occur even with low doses, toxicity may be exacerbated with concurrent administration with NSAID, patients must be adequately counseled on drug interactions and serious side effects before initiating baclofen, and patients and physicians must be aware of potential withdrawal symptoms following an abrupt cessation or dose reduction.
